# Supplementary material for: Comparison of Nonlinear Growth Models to Estimate Growth Curves in Kivircik Sheep under a Semi-Intensive Production System
Source: Animals (Basel). 2023 Jul 21;13(14):2379. doi: 10.3390/ani13142379 (PMC10376270; doi:10.3390/ani13142379)
Supplement: Supplementary file 1 [file animals-13-02379-s001.zip › animals-2453449-supplementary.pdf]

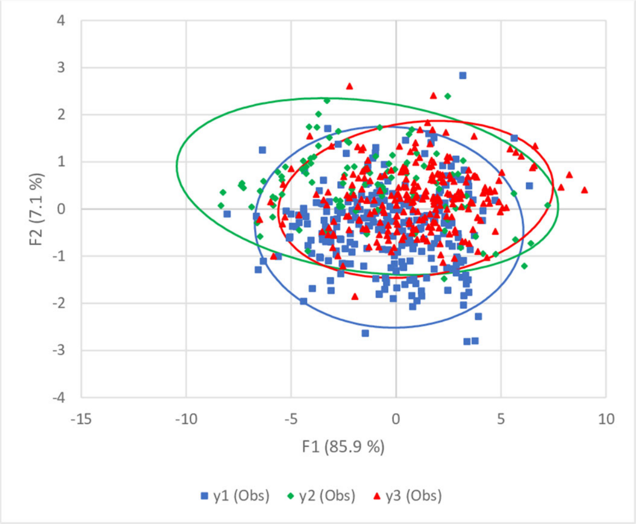

Figure S1: Scatter plot of the first two principal components for the age–weight observations grouped by the year of birth. The ellipses represent the 95% confidence intervals for 2014-, 2015-, 2016-born lambs, reported as y1, y2, and y3, respectively.

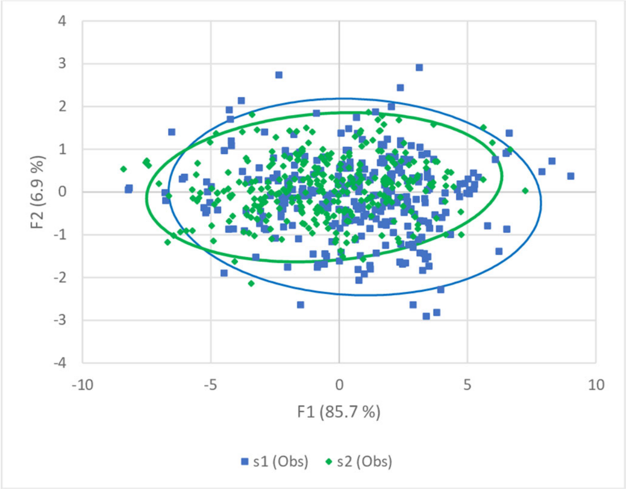

Figure S2: Scatter plot of the first two principal components for the age–weight observations grouped by sex. The ellipses represent the 95% confidence intervals for male and female lambs, reported as s1 and s2, respectively

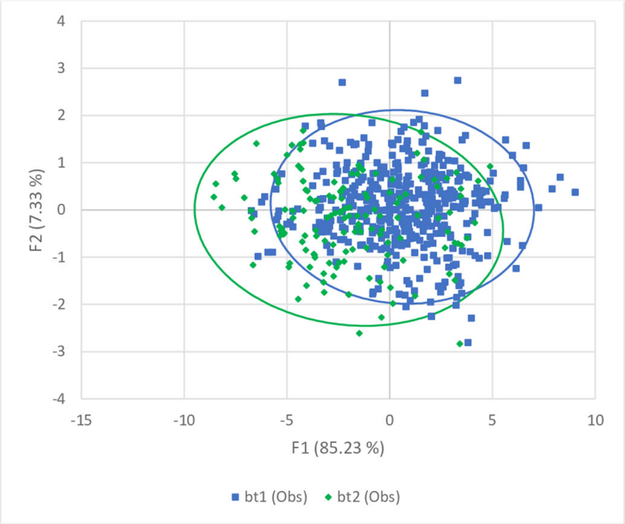

Figure S3: Scatter plot of the first two principal components for the age–weight observations grouped by birth type. The ellipses represent the 95% confidence intervals for single- and twin-born lambs, reported as bt1 and bt2, respectively.

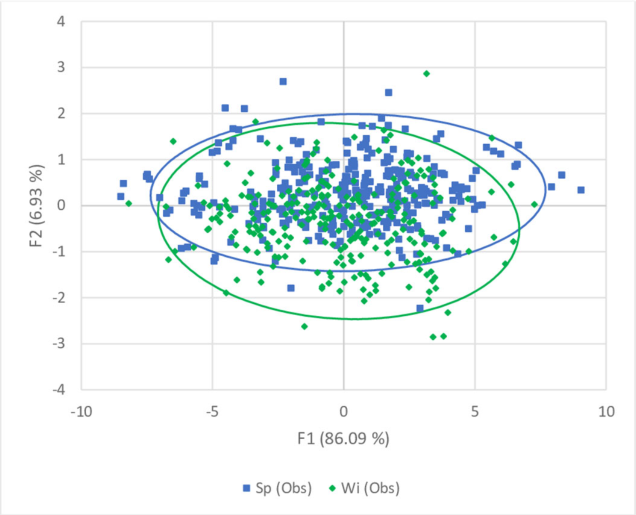

Figure S4: Scatter plot of the first two principal components for the age–weight observations grouped by season of birth. The ellipses represent the 95% confidence intervals for spring- and winter-born lambs, reported as Sp and Wi, respectively.
